# Moisture migration analysis of Chinese naked oat during different storage conditions by sorption isotherm model and low‐field NMR

**DOI:** 10.1002/fsn3.1461

**Published:** 2020-02-17

**Authors:** Lifang Cao, Bowen Li, Nan Zhao, Huan Li, Yanfeng Wang, Xing Yu, Xin Huang

**Affiliations:** ^1^ Yellow River Conservancy Technical Institute Kaifeng China; ^2^ Bioenergy and Environment Science & Technology Laboratory College of Engineering China Agricultural University Beijing China; ^3^ Key Laboratory of Clean Production and Utilization of Renewable Energy Ministry of Agriculture and Rural Affairs China Beijing China; ^4^ National Engineering Laboratory for Crop Efficient Water Use and Disaster Mitigation Key Laboratory of Dryland Agriculture and Key Laboratory for Prevention and Control of Residual Pollution in Agricultural Film Ministry of Agriculture and Rural Affairs Beijing China; ^5^ Institute of Environment and Sustainable Development in Agriculture Chinese Academy of Agricultural Sciences Beijing China

**Keywords:** adsorption, bound water, crop, desorption, free water, moisture content

## Abstract

Moisture migration is considered to be one of the most important influencer on crop quality during storage, which is easily affected by storage conditions, such as ambient humidity and temperature. The aim of this work was to determine the effect of storage condition on moisture content of Chinese naked oat by simulating 9 equilibrium relative humidity (ERH) and 5 temperatures. The equilibrium moisture content (EMC) of dry sample was achieved by adsorption, while EMC of wet one was achieved by desorption. EMC of oat increased with the increase in ERH and decreased when temperature increased. The sorption isotherm was a typical “S” shape and fitted using current EMC/ERH models. Modified Chung–Pfost (MCPE) model was the most suitable for describing the dynamic sorption process of Chinese naked oat during storage with a ERH range from 10% to 90%. There was an obvious hysteresis between adsorption and desorption isotherms, whose range decreased with the increase of temperature. High temperature accelerated moisture migration by increasing the hydrophilicity of oat surface. Moreover, dynamic moisture migration was imaged by low‐field nuclear magnetic resonance (NMR), showing that moisture migrated between ambient environment and oat mainly through endosperm where most moisture accumulated. During sorption, free water migrated firstly, followed by bound water and the change in content of bound water was more stable than that of free water. The results of this study can provide a useful information for future work on quality control of oat during storage.

## Graphical abstract

We determined the dynamic sorption of oat under different ambient humidity and temperatures by isotherm model. We imaged moisture migration during adsorption and desorption processes. We analyzed the changes in content of bound water and free water during storage.

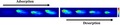

## 1 INTRODUCTION

Oat, an important coarse cereal, is widely used in food, medicine, feed, and economy fields (Zhao, Fu, Li, Wang, & Chen, 2017). It has a relative low requirement for production condition and self‐reproduction ability, which can grow in nutrient‐poor and low rainfall area (Ren et al., 2014). Oat has been widely planted in many countries, including China and Russia, whose production has remained at more than 100 million tons over past five years, only inferior to wheat, corn, rice, and barley (Liu, Hsieh, Heymann, & Huff, 2000; Romaní, Tomaz, Garrote, Teixeira, & Domingues, 2016). Oat itself has low sugar and high energy as well as many nutritional components and biologically active substances, which is often used for preventing diabetes and other diseases (Dongowski et al., 2005; Du et al., 2008; Yang, Wang, & Chen, 2017).

Moisture content is considered as a significant influencer on oat quality (Zhao et al., 2018). Generally, oat needs to be stored before end‐user processing and it will be inactive (moisture adsorption) or mold (moisture desorption) easily during storage because of the variance between ambient humidity and initial moisture content of oat, directly causing a benefit loss (Bahloul, Boudhrioua, & Kechaou, 2008). The same issues were also found in wood and other biological products (Simo‐Tagne, Rémond, Rogaume, Zoulalian, & Bonoma, 2016b; Simo‐Tagne, Rémond, Rogaume, Zoulalian, & Perre, 2016a). Therefore, it is necessary to understand the dynamic relationship between equilibrium relative humidity (ERH) and equilibrium moisture content (EMC) of oat. This relationship is called as EMC/ERH isotherm, which is often affected by ambient temperature (Mehkov & Dinkov, 1999). The isotherm is closely related with moisture migration, determining the final moisture contents of crops during storage (Tsami, 1991). Currently, there are lots of studies about using sorption isotherm to describe the dynamic migration of crops during different environmental conditions (Bartosik & Maie, 2007; Basunia & Abe, 2005; Hulasare & Habok, 2001). Sun (1998, 1999) fitted EMC/ERH data from wheat, corn, and rice, finding that modified Oswin model, modified Henderson model, and modified Strohman–Yoerger model were suitable for wheat, corn, and rice, respectively. San Martin et al. (San Martin, Mate, Fernandez, & Virseda, 2007) evaluated isotherm models of rough rice through some parameters such as mean relative standard error. The best model for describing the relationships between EMC and ERH of triticale seed or mung bean was modified Chung–Pfost model (Agha, Bucklin, Teixeira, Lee, & Blount, 2014; Chowdhury, Huda, Hossain, & Hassan, 2006). There are hundreds of theoretical, semi theoretical, and empirical models describing sorption isotherm of crops (Blahovec, 2005); however, few studies focus on oat.

Surface wettability property of seed coat is another important parameter to evaluate the performance of moisture migration (Koizumi & Kano, 2016). This property is widely measured by optical contact angle (0°–180°) measuring instrument with a liquid drop method (Roman‐Gutierrez, Sabathier, Guilbert, Galet, & Cuq, 2003). The smaller contact angle is, the stronger hydrophilicity (<90º) is and the easier moisture migrates is (Beatty & Smith, 2010). This angle is affected by ambient temperature. Moreover, low‐field 1H nuclear magnetic resonance (NMR) is often used for imaging dynamic moisture migration of crops (Song, Yang, Wang, Pan, & Ren, 2015; Yao et al., 2015). The contents of bound water and free water can be also determined by this instrument (Chen et al., 2017).

The aim of this work was to simulate different storage conditions and investigate the effect of ERH and temperature on EMC of oat in both adsorption and desorption models. The sorption isotherms were fitted by 9 models to select the best model for describing the relationships between EMC and ERH of oat. The surface wettability of oat was determined by measuring contact angle at different temperatures (10, 15, 20, 25, and 30°C). Low‐field NMR was used to analyze the changes in contents of bound water and free water as well as imaging the dynamic process of moisture migration between ambient environment and oat.

## 2 MATERIALS AND METHODS

### 2.1 Sample preparation

A typical Chinese naked oat of Jizhangyou 4 cultivar, grown in the dawn area of Inner Mongolia of Northern China, was used in this study. In order to ensure sorption experiments conducted well, the initial moisture content of adsorption sample must be lower than the lower limit of EMC after water intake, while the initial moisture content of desorption sample must be higher than the upper limit of EMC after water loss (Li, 2012; Miranda et al., 2012). Therefore, fresh oat was dried to reduce its initial moisture content to a value below 5.0% (w.b.) in an electric thermostatic drying oven (Yiheng Scientific Laboratory Instrument, Shanghai, China) as adsorption sample (AS), while fresh oat was soaked in water to increase its high initial moisture content to a value above 30.0% (w.b.) as desorption sample (DS) (Li, Cao, Wei, Feng, & Wang, 2011). It was also worth noting that the initial moisture content of oat was controlled at an appropriate value, under which oat quality was not destroyed. All samples were then sealed in polyethylene bag with headspace air removed and stored in refrigerator at 4°C before experiment (Santalla & Mascheroni, 2003). Generally, the size and mass of single oat are much small and its moisture content change is hard to control and measured. In this study, 5 g AS or DS composed of multiple oats was used for each sorption experiment and all results were the average values of 5 g oat samples. This avoided the impacts of inhomogeneous repartitions of different oat samples on result precisions. The initial moisture content was measured by http://www.lisservice.com/shop/mettler-hb43s-halogen-moisture-balance-p-188.html?zenxml:id=3oksvogf42fkhj7ahqcr0g1r25 (HB43‐S, Mettler Toledo, USA), which was 2.38% and 33.80% for 5 g AS and DS, respectively.

### 2.2 Adsorption/desorption experiment

5 g AS and DS were placed in a rapid glass dryer when 9 saturated salt solutions (Table 1) were poured into 3 cm beneath the samples. The moisture content of samples gradually reached equilibrium by adsorption or desorption under different ERH produced by different saturated salt solutions in confined space (Lan & Kunze, 1996). Rapid glass dryer was placed in illumination incubator (GP‐01, Huangshi Hengfeng Medical Instrument Co., Ltd., Hubei), and temperature was controlled by constant temperature system (10, 15, 20, 25, 30 ºC). Samples were weighed by using a balance (AB135‐S, Mettler Toledo, USA) once a day until the variance between the two continuous weighing was not more than 0.01 g.

**Table 1:** ERH produced by some saturated salt solutions

Saturated salt solution	ERH (%) Temperature (°C)				
	10	15	20	25	30
Lithium chloride	11.29	11.30	11.31	11.30	11.28
Potassium acetate	23.38	23.40	23.11	22.51	21.61
Magnesium chloride	33.47	33.30	33.07	32.78	32.44
Potassium carbonate	43.14	43.15	43.16	43.16	43.17
Magnesium nitrate	58.86	57.36	55.87	54.38	52.89
Copper chloride	68.40	68.40	68.30	67.00	66.50
Sodium chloride	75.67	75.61	75.47	75.29	75.09
Potassium chloride	86.77	85.92	85.11	84.34	83.62
Potassium nitrate	95.96	95.41	94.62	93.58	92.31

### 2.3 Sorption model

EMC data of oat obtained at different ERH and temperatures were fitted by 9 commonly cited sorption isotherm models including modified Henderson (MHE), modified Chung–Pfost (MCPE), modified Halsey (MHAE), modified Oswin (MOE), modified Guggenheim–Anderson–de Boer (MGAB), Chen–Clayton (CCE), modified Strohman–Yoerger (SYE), modified BET (BET), and Wu equation (CAE) (Table 2) (Bonner & Kenney, 2013; Li, 2012). Model parameters were calculated using logistic regression method of SPSS 20 (Inc Chicago, SPSS, USA), and sorption isotherm curve was drawn by Origin 8.0 (OriginLab, Origin, USA).

**Table 2:** Nine common EMC/ERH sorption isotherm models used for fitting the experimental data

Model	Equation*
MHE	$M={\left[-\frac{\ln\left(1-h_{r}\right)}{C_{1}\left(C_{2}+T\right)}\right]}^{\frac{1}{C_{3}}}]]>$
MCPE	$M=-\frac{1}{C_{3}}\ln\left[-\frac{\left(T+C_{2}\right)lnh_{r}}{C_{1}}\right]]]>$
MHAE	$M={\left[-\frac{\exp\left(C_{1}+C_{2}T\right)}{lnh_{r}}\right]}^{\frac{1}{C_{3}}}]]>$
MOE	$M=\frac{C_{1+}C_{2}T}{{\left(\frac{1}{h_{r}}-1\right)}^{\frac{1}{C_{3}}}}]]>$
MGAB	$M=\frac{C_{1}C_{2}\left(\frac{C_{3}}{T}\right)h_{r}}{\left(1-C_{2}h_{r}\right)\left(1-C_{2}h_{r}+\frac{C_{3}}{T}C_{2}h_{r}\right)}]]>$
CCE	$M=\frac{1}{-C_{3}{\left(T+273.15\right)}^{C_{4}}}\ln\left[\frac{{\left(T+273.15\right)}^{C_{2}}lnh_{r}}{-C_{1}}\right]]]>$
SYE	$h_{r}=\exp\left[C_{1}\exp\left(-C_{2}M\right)lnP_{s}-C_{3}\exp\left(-C_{4}M\right)\right]]]>$
BET	$M=\frac{\left(C_{1}+C_{2}T\right)C_{3}h_{r}}{\left(1-h_{r}\right)\left(1-h_{r}+C_{3}h_{r}\right)}\left(h_{r}<50\%\right)]]>$
CAE	$h_{r}=\exp\left\{\frac{\frac{C_{5}}{222}\left[\exp\left(\frac{C_{3}-M}{C_{1}}\right)-\exp\left(\frac{C_{4}-M}{C_{2}}\right)\right]\left(1737.1-\frac{474242}{273+T}\right)+C_{5}\left[1-\exp\left(\frac{C_{3}-M}{C_{1}}\right)\right]+202}{87.72}\right\}]]>$

* *M* is equilibrium moisture content (%); *h_r_* is environmental equilibrium relative humidity (%); C_1_, C_2_, C_3_, C_4_, and C_5_ are equation parameters; *T* is temperature (°C); and *P_s_* is saturated vapor pressure (MPa).

There were four quantitative indicators used to compare performance of sorption isotherm model to select the best model for oat in this study, including coefficient of determination (*R^2^*), residual sum of squares (*RSS*), standard error of the estimate (*SEE*), and mean relative deviation (*MRD*) (Miranda et al., 2012).

(1) $$R^{2}=1-\frac{\sum_{i=1}^{n}{\left(M_{i}-M_{\mathrm{pi}}\right)}^{2}}{\sum_{i=1}^{n}{\left(M_{i}-M_{\mathrm{mi}}\right)}^{2}}$$

(2) $$\text{RSS}=\sum_{i=1}^{n}{\left(M_{i}-M_{\mathrm{pi}}\right)}^{2}$$

(3) $$\text{SE}=\sqrt{\frac{\sum_{i=1}^{n}{\left(M_{i}-M_{\mathrm{pi}}\right)}^{2}}{n-1}}$$

(4) $$\text{MRE}\%=\frac{100}{n}\sum_{i=1}^{n}\left|\frac{M_{i}-M_{\mathrm{pi}}}{M_{i}}\right|$$

where *M_e_* is experimental moisture content of oat (%, w.b.), *M_p_* is predicted moisture content (%, w.b.), *M_m_* is mean moisture content (%, w.b.), and *n* is the number of data.

*R^2^* represents the variability between experimental and predicted data, *RSS* and *SEE* measure the accuracy of model, and *MRD* is used to describe the goodness of fitting model because *SEE* cannot provide a clear visualization of the goodness of fit (Aviara, Ajibola, & Dairo, 2003). The model with the lowest *RSS*, *SE,* and *MRE* values as well as greatest *R^2^* value was considered as the best fitting model in this study.

### 2.4 Determination of surface wettability

The surface wettability of oat was measured directly using an optical contact angle measuring instrument (JC2000DM, Powereach Co., Ltd., Shanghai, China) with a goniometer, a CCD camera according to the sessile drop method (Blancher, Morel, Gastaldi, & Cuq, 2007; Roman‐Gutierrez et al., 2003). Oat was placed on a platform, and dorsal side was chosen as the testing surface. Working temperature was set as 10, 15, 20, 25, and 30°C, respectively. The water drop was captured sequentially after contacting with the oat surface, and the corresponding contact angle was calculated by an average value between advancing and retreating angles (Beatty & Smith, 2010).

### 2.5 Low‐field NMR

Low‐field 1H NMR measurements were performed using a 12.2 MHz pulsed NMR analyzer (NMI20‐015V‐I, Niumag Co., Ltd., Suzhou, China) according to the method reported by Song et al. (2015). The probe of this analyzer (5 mm in diameter) was inserted into sample to measure the spin–spin relaxation time (*T_2_*) and corresponding signal amplitude based on the Carr–Purcell–Meiboom–Gill (CPMG) sequence at temperature of 32°C. The pulse parameters of CPMG sequence were as follows: 90° pulse radio frequency pulse width = 4 μs, 180° pulse radio frequency pulse width = 8 μs, repeated sampling waiting time = 5,000 ms, echo time = 0.150 ms, echo number = 6,000, and repeated sampling frequency = 64. *T_2_* spectra of bound water and free water were 0.1–10 ms and 10–1000 ms, respectively, and the corresponding area under the curve of the peak with *T_2_* range was proportional to the content of bound water or free water (Song et al., 2015).

After *T_2_* measurement, 2D proton density image of sample was captured from transverse section (Chen et al., 2017; Yao et al., 2015). The pulse parameters were as follows: field of view (FOV) read = 80 mm, FOV phase = 80 mm, repetition time = 120 ms, echo time = 5.885 ms, slices = 1, slice width = 7 mm, and averages = 128. The size of image was 192 × 256 pixels.

## 3 RESULTS AND DISCUSSIONS

### 3.1 Determination of EMC

An error caused by air flow, existing between experimental value of EMC and actual one, was ignored in this study. The average EMC of oat obtained from adsorption or desorption processes at 9 ERH and 5 temperatures is presented in Table 3, which showed that EMC of oat correlated closely with temperature and ERH. This value in general increased with the increase of ERH, while decreased when temperature increased. There was also an obvious hysteresis between EMC values from adsorption and desorption process, which was called as adsorption hysteresis, that is, EMC values from adsorption were always less than desorption at all ERH. This hysteresis can reflect the potentials of structural and conformational rearrangements of oat (Al‐Muhtaseb, Mcminn, & Magee, 2004; Santalla & Mascheroni, 2003). The smaller hysteresis is, the stronger potential of oat rearrangement after drying or soaking is. This hysteresis was reduced significantly by increasing temperature because high temperature improved moisture migration including enhancing moisture uptake during adsorption and moisture loss during desorption. This hysteresis was usually reported in sorption researches of crops, such as wheat (Li et al., 2011), sunflower kernel (Santalla & Mascheroni, 2003), and quinoa seed (Miranda et al., 2012). It was worth noting that sample preparation was also a contributor to the presence of hysteresis in this study because there were some inevitable differences between AS and DS. Therefore, we will use a two‐cycle sorption methods to study oat hysteresis in the further research where only one sample desorbs water first under drying environment and then adsorbs under wet environment until reaching EMC (Bonner & Kenney, 2013).

**Table 3:** EMC of oat obtained from adsorption and desorption at different storage conditions

Environmental conditions		Equilibrium moisture content (%)		Hysteresis (%)	Environmental conditions		Equilibrium moisture content (%)		Hysteresis (%)
Temperature (°C)	Relative humidity (%)	Adsorption	Desorption		Temperature (°C)	Relative humidity (%)	Adsorption	Desorption	
10	11.29	8.45	8.97	0.52	25	11.30	6.23	7.16	0.93
	23.38	9.80	10.13	0.33		22.51	7.96	9.06	1.10
	33.47	9.42	11.28	1.9		32.78	8.97	10.37	1.41
	43.14	11.77	13.17	1.4		43.16	10.72	11.61	0.88
	58.86	14.12	14.56	0.44		54.38	11.42	12.50	1.08
	68.40	15.78	17.09	1.31		67.00	13.76	14.90	1.14
	75.67	15.83	17.57	1.75		75.29	15.14	15.83	0.69
	86.77	18.84	20.65	1.81		84.34	17.61	17.88	0.27
	95.96	24.76	25.15	0.39		93.58	22.05	22.31	0.26
15	11.30	6.04	7.84	1.80	30	11.28	5.86	6.58	0.72
	23.40	7.84	9.62	1.77		21.61	7.63	8.57	0.94
	33.30	9.32	10.96	1.65		32.44	9.01	9.82	0.81
	43.15	10.63	12.17	1.54		43.17	10.50	11.45	0.95
	57.36	12.27	14.04	1.77		52.89	11.25	12.22	0.97
	68.40	14.25	15.94	1.68		66.50	13.60	14.16	0.56
	75.61	16.27	17.13	0.86		75.09	15.12	15.84	0.72
	85.92	18.80	19.61	0.81		83.62	17.35	17.57	0.22
	95.41	23.45	26.97	3.52		92.31	20.59	20.91	0.33
20	11.31	6.38	77.19	0.82					
	23.11	8.63	9.14	0.51					
	33.07	9.17	10.60	1.43					
	43.16	11.01	12.58	1.57					
	55.87	12.30	13.72	1.41					
	68.30	14.02	15.36	1.34					
	75.47	15.45	16.75	1.29					
	85.11	18.09	18.87	0.79					
	94.62	23.09	23.60	0.52					

### 3.2 Mathematical modeling of EMC/ERH isotherm

The modeling parameters and error indicators of fitting ERH/EMC of oat during adsorption and desorption at 5 temperatures are presented in Tables 4 and 5. As reported by Li, Wang, Jiang, and Li (2016), the fitting performance of model is generally evaluated by comparing *MRE* value and this model is considered as an excellent one when its *MRE* is less than 10%. Results showed that *MRE* values of all models were less than 10% with the exception of CAE model in this study, meaning that CAE was not suitable for fitting sorption data of oat. *R^2^* values of all models were higher than 0.95 with the exception of BET model; therefore, this model was also removed. The highest *R^2^* value was found in MCPE model, followed by SYE and CCE for adsorption, while the highest *R^2^* value was obtained from MCPE, followed by SYE and MHE for desorption. Moreover, MCPE model also had the lowest *RSS* and *SE* values, while CAE and SYE models had highest values.

**Table 4:** The estimation of parameters of 9 isotherm models for fitting adsorption data

Model	Pertinent parameters					R^2^	Error indicators		
	C_1_	C_2_	C_3_	C_4_	C_5_		RSS	SE	MRE%
MHE	0.858	90.600	2.302			0.981	0.002	0.007	4.92
MCPE	1259.000	125.700	22.070			0.996	4.901E‐4	0.003	2.93
MHAE	−7.619	−0.009	3.406			0.958	0.005	0.010	7.65
MOE	0.122	−3.630E‐4	4.035			0.982	0.002	0.008	4.57
GAB	0.078	0.694	778.300			0.985	0.002	0.006	3.78
CCE	1.734	−0.300	0.032	1.153		0.989	0.001	0.005	3.69
SYE	0.165	11.810	8.218	21.270		0.995	0.017	0.019	7.77
BET	0.066	−1.903E‐4	138.1			0.915	0.001	0.005	4.88
CAE	0.351	0.286	1.381	1.122	4.691	0.957	0.147	0.058	11.46

**Table 5:** The estimation of parameters of 9 isotherm models for fitting desorption data

Model	Model parameters					R^2^	Error parameters		
	C_1_	C_2_	C_3_	C_4_	C_5_		RSS	SE	MRE%
MHE	2.927	39.15	2.703			0.996	4.050E‐4	0.003	3.20
MCPE	648.500	30.990	23.040			0.999	1.458E‐4	0.002	2.04
MHAE	−7.253	−0.024	3.530			0.969	0.004	0.009	5.88
MOE	0.144	−0.001	4.293			0.991	0.001	0.005	3.20
GAB	0.094	0.606	747.400			0.987	0.002	0.006	3.13
CCE	1.039	−0.438	0.019	1.247		0.988	0.001	0.006	2.76
SYE	1.402	15.930	12.500	21.170		0.997	0.010	0.015	4.07
BET	0.082	−5.285E‐4	192.500			0.955	3.042E‐4	0.004	3.13
CAE	0.357	0.259	2.334	1.698	0.351	0.964	0.121	0.052	9.43

After comprehensive evaluation, the fitting performances of adsorption data were ranked as follows: MCPE > CCE>GAB > MOE>MHE > MHAE>BET > SYE>CAE, while the fitting performances of desorption data were ranked as follows: MCPE > MHE>MOE > CCE>BET > GAB>MHAE > SYE>CAE. MCPE model was selected as the best one for fitting the EMC/ERH data of oat during both adsorption and desorption, which was the most suitable for describing sorption process and moisture migration of oat during storage humidity ranging from 10% to 90%. This model of this work can enable researchers to accurately predict EMC value of oat under a known storage conditions (ERH and temperature), which is good for safe storage (Zomorodian, Kavoosi, & Momenzadeh, 2011). It was worth noting that the goodness of fit of desorption models was always better than adsorption one due to the presence of adsorption hysteresis.

### 3.3 Simulation of oat sorption by selected model

**Figure 1:**
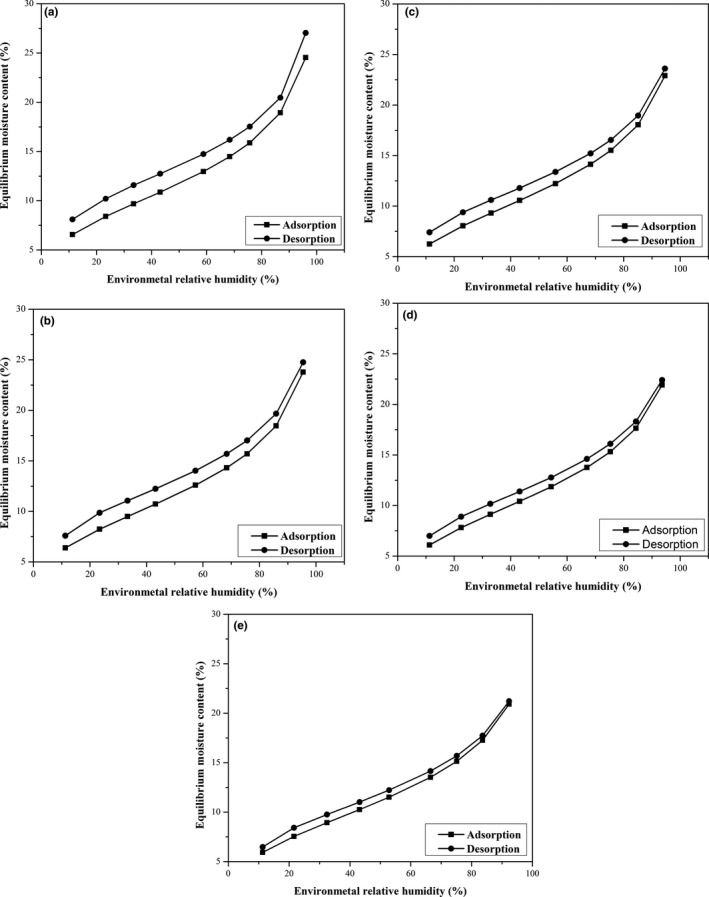
The predicted sorption isotherm curve of oat by MCPE model at (a) 10, (b) 15, (c) 20, (d) 25, and (e) 30°C

Figure 1 shows that sorption isotherm curve of oat was a typical “S” shape at whatever the temperature was. At constant temperature, EMC value increased with the increase in ERH. This growth was relatively smooth when ERH ranged from 30% to 70%, while rapid growth was found at the ERH range of 10%–30% or 70%–90%. High temperature reduced EMC value of oat when ERH was constant, and this reduction effect on EMC was more obvious when ERH was less than 80%. Adsorption isotherm curves always lied below desorption ones because of the presence of adsorption hysteresis which existed over the whole ERH range. This hysteresis was caused by a thermodynamically irreversible process during sorption, whose extent was affected significantly by ERH and temperature (Li, 2012; Sun & Woods, 1994). Polar sites in the molecular structure of wet oat were entirely occupied by moisture, causing a close adhesion between molecules and their moisture holding sites and directly reducing moisture migration from oat into ambient environment during desorption (Sinija & Mishra, 2008). The hysteresis was relatively obvious at the ERH range of 20%–70% and weak at the RH range of 10%–20% or 70%–90%, whose span was also reduced by increasing ambient temperature.

### 3.4 Surface wettability

**Figure 2:**
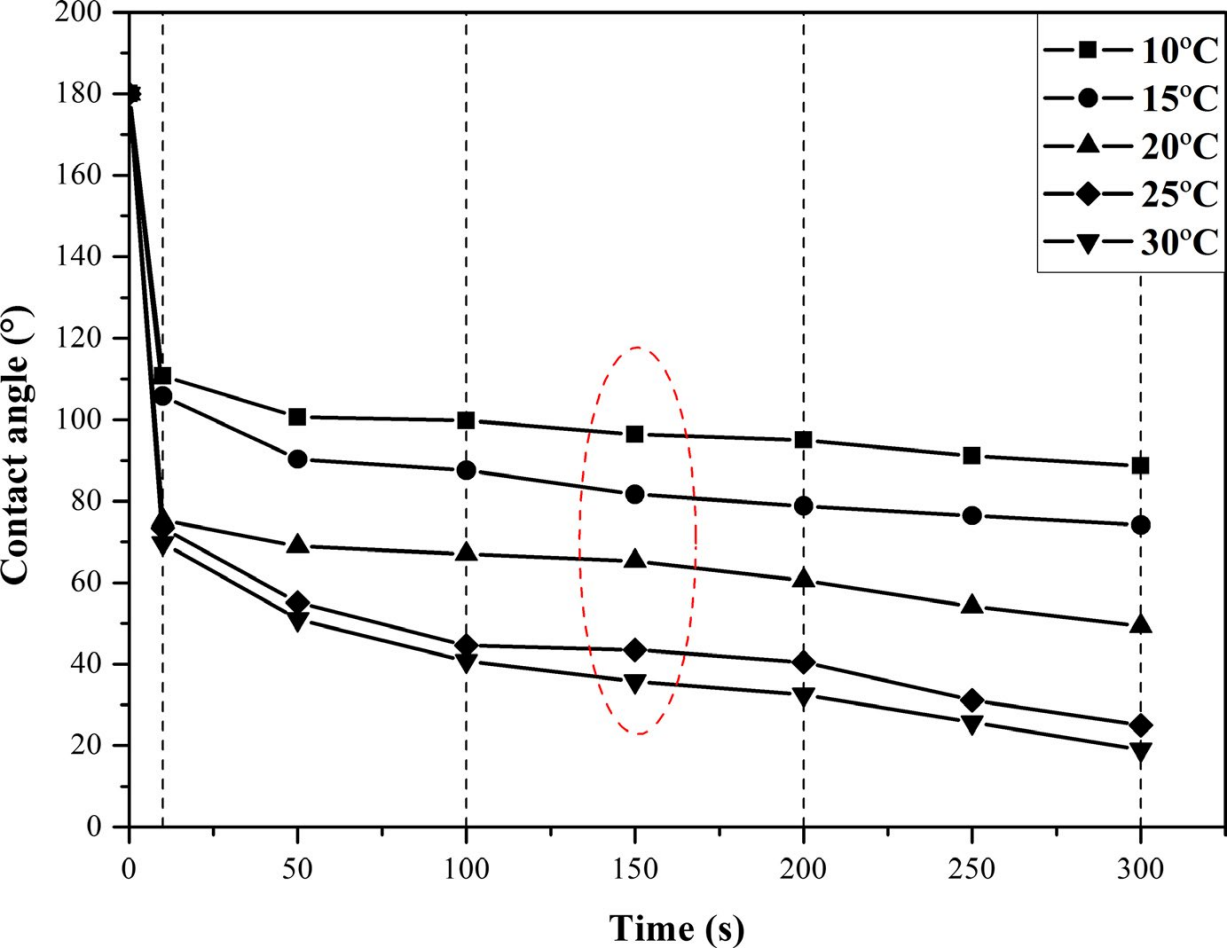
Changes in contact angle of water drop deposited on oat surface at temperatures of 10, 15, 20, 25, and 30°C

The surface wettability of oat was determined by measuring contact angle of a water drop deposited on oat surface. The typical change in contact angle as a function of time at different temperatures is presented in Figure 2. This curve was divided into four regions. In the first region (0–10 s), contact angle decreased sharply from 180° to a relatively low value because the gravity of water drop caused a rapid spreading on oat surface in a short time. In the second region (10–100 s), contact angle decreased slowly until reaching a relatively stable value, meaning water started to migrated to the inner of oat (Roman‐Gutierrez et al., 2003). In the third region (100–200 s), there was almost no change in contact angle because oat was saturated with moisture. The equilibrium value of contact angle at over 150 s was used to characterize the hydrophobicity/hydrophilicity of oat surface, which was 96°, 82°, 65°, 44°, and 37° for 10, 15, 20, 25, and 30°C, respectively. In the fourth region (200–300 s), a slight decrease was found in contact angle due to evaporation of water drop (Beatty & Smith, 2010). This decreasing trend was more obvious by increasing ambient temperature because high temperature enhanced evaporation of water drop. The magnitude of curve decreased with the increase in temperature, meaning that high temperature increased hydrophilicity of oat surface and accelerated moisture migration through seed coat of oat.

### 3.5 Dynamic moisture migration

**Figure 3:**
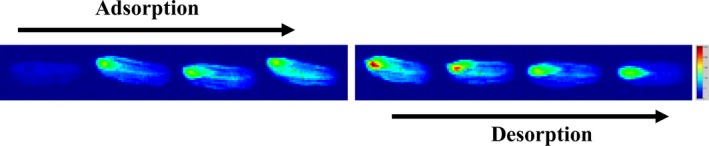
Pseudocolor of 2D proton density images of oat during adsorption and desorption

The dynamic moisture migrations of AS (2.38%) adsorption and DS (33.8%) desorption imaged by low‐field ^1^H NMR are shown in Figure 3. Color of oat in these images changed from blue to red, meaning that moisture content of oat increased (Lu, Guo, Li, & Ming, 2017). Moisture migrated mainly through endosperm, most of which accumulated in endosperm and surrounding regions. This was because endosperm was more hydrophilic than other part of oat and moisture migration was easier when going through it (Yao et al., 2015). During adsorption, moisture migrated from ambient environment to endosperm of oat through seed coat and gradually diffused to whole oat, while during desorption, moisture loss occurred first in nonendosperm region of oat, followed by endosperm. When reaching EMC by desorption, there was still a certain amount of moisture stored in endosperm because most moisture in endosperm was bound water (Song et al., 2015).

**Figure 4:**
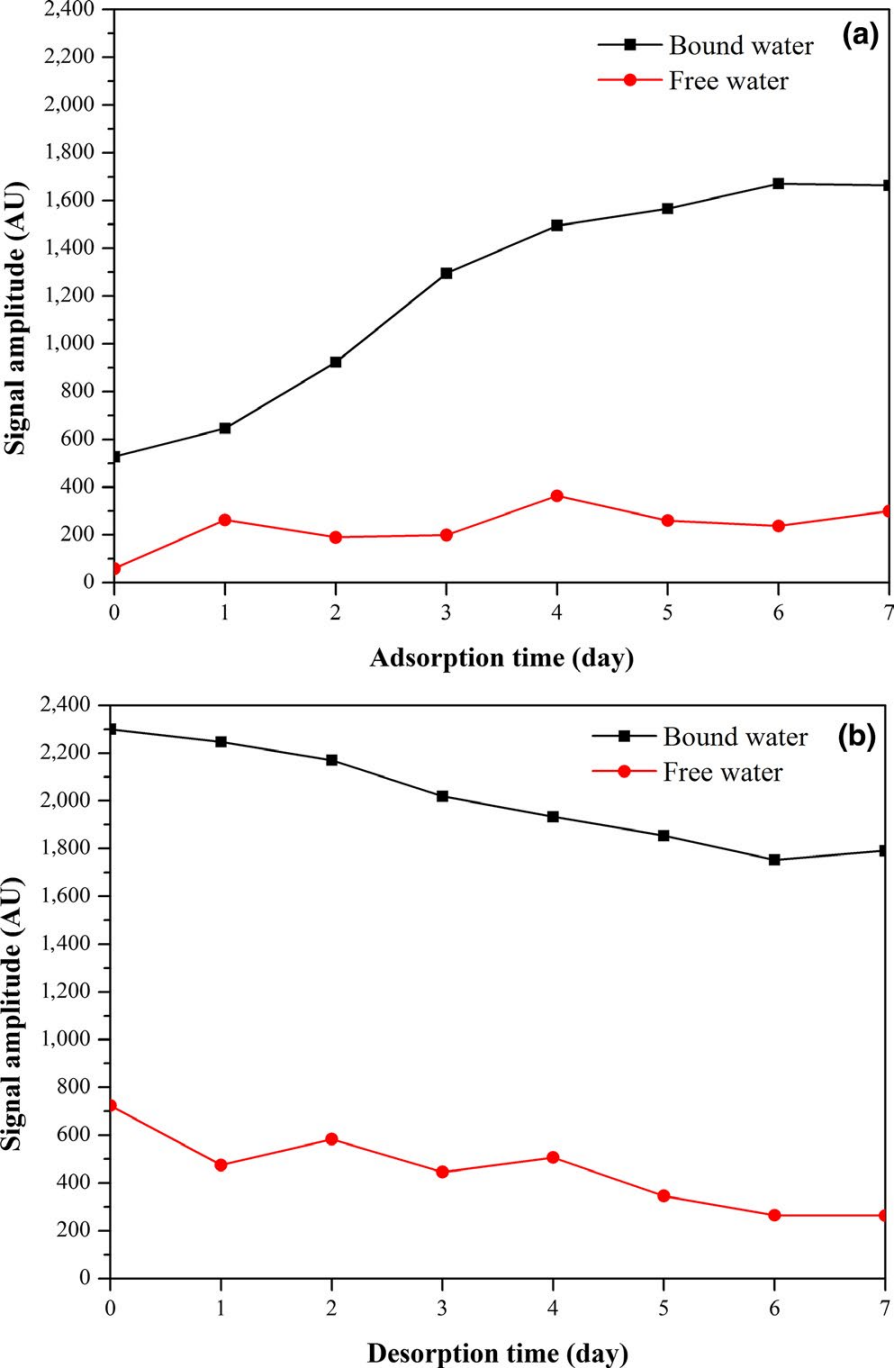
Changes in signal amplitudes of bound water and free water with oat (a) adsorption and (b) desorption times

Change in signal amplitude of oat during adsorption or desorption is presented in Figure 4 as a function of sorption time. Generally, signal amplitude of oat is positively correlated with its moisture content. During early stage of adsorption, most moisture adsorbed in oat was free water, while the increase of bound water was relatively slow. Then with the reactions of free water and nutrients of oat, such as protein, starch, and carbohydrate, the adhesion of moisture content with oat was improved, causing a transition from free water to bound water (Song et al., 2015). Therefore, the content of bound water started to increase rapidly during this stage and gradually tended to an equilibrium value due to moisture saturation of oat. Similarly, moisture loss of free water was more obvious than bound water during the early stage of desorption, while the decline rate of bound water content gradually increased until reaching an equilibrium value with the process of desorption. The equilibrium contents of bound water and free water after desorption were higher than adsorption ones because of adsorption hysteresis. Moreover, the change in content of bound water was more stable than that of free water.

## 4 CONCLUSION

Moisture content and status (free water and bound water) are the key factors on affecting the quality of Chinese naked oat during storage, which changed frequently with the process of moisture migration caused by adsorption and desorption. This work simulated different storage conditions (5 temperatures and 9 ERH) and determined their effects on EMC of oat. EMC value increased with the increase in ERH and decreased when temperature increased. This value achieved from desorption was always higher than adsorption because of a hysteresis effect which was weaken by increasing ambient temperature. Oat sorption isotherm was a typical “S” shape, which was fitted well by MCPE model. High temperature increased the surface wettability of seed coat and improved its hydrophilicity, causing an easier moisture migration between ambient environment and oat. During sorption, endosperm was the main “bridge” of moisture migration, where most moisture accumulated. The change in bound water content was more stable than free water. This work can provide an effective method for analyzing moisture migration during oat sorption, as well as the data support for seeking the best storage condition.

## CONFLICT OF INTEREST

The authors declare that they have no competing financial interests or personal relationships that could have appeared to influence the work reported in this paper.

## ETHICAL APPROVAL

The authors declare that this study did not involve human or animal subjects and human and animal testing are unnecessary in this study.
